# Clinical, Biological and Genetic Analysis of Anorchia in 26 Boys

**DOI:** 10.1371/journal.pone.0023292

**Published:** 2011-08-10

**Authors:** Raja Brauner, Mathieu Neve, Slimane Allali, Christine Trivin, Henri Lottmann, Anu Bashamboo, Ken McElreavey

**Affiliations:** 1 Université Paris Descartes and AP-HP, Hôpital Bicêtre, Unité d'Endocrinologie Pédiatrique, Le Kremlin Bicêtre, France; 2 Assitance publique-Hopitaux de Paris, Hôpital Necker-Enfants Malades, Service d'explorations fonctionnelles, Paris, France; 3 Assitance publique-Hopitaux de Paris, Hôpital Necker-Enfants Malades, Service de chirurgie viscérale pédiatrique, Paris, France; 4 Human Developmental Genetics Unit, Institut Pasteur, Paris, France; University of Muenster, Germany

## Abstract

**Background:**

Anorchia is defined as the absence of testes in a 46,XY individual with a male phenotype. The cause is unknown.

**Methods:**

We evaluated the clinical and biological presentation, and family histories of 26 boys with anorchia, and sequenced their *SRY*, *NR5A1*, *INSL3*, *MAMLD1* genes and the *T222P* variant for *LGR8*.

**Results:**

No patient had any associated congenital anomaly. At birth, testes were palpable bilaterally or unilaterally in 13 cases and not in 7; one patient presented with bilateral testicular torsion immediately after birth. The basal plasma concentrations of anti-Müllerian hormone (AMH, n = 15), inhibin B (n = 7) and testosterone (n = 19) were very low or undetectable in all the patients evaluated, as were the increases in testosterone after human chorionic gonadotropin (hCG, n = 12). The basal plasma concentrations of follicle stimulating hormone (FSH) were increased in 20/25, as was that of luteinising hormone in 10/22 cases. Family members of 7/26 cases had histories of primary ovarian failure in the mother (n = 2), or sister 46,XX, together with fetal malformations of the only boy with microphallus and secondary foot edema (n = 1), secondary infertility in the father (n = 2), or cryptorchidism in first cousins (n = 2). The sequences of all the genes studied were normal.

**Conclusion:**

Undetectable plasma concentrations of AMH and inhibin B and an elevated plasma FSH, together with 46,XY complement are sufficient for diagnosis of anorchia. The hCG test is unnecessary. *NR5A1* and other genes implicated in gonadal development and testicle descent were not mutated, which suggests that other genes involved in these developments contribute to the phenotypes.

## Introduction

Anorchia, or embryonic testicular regression (vanishing testis syndrome), is defined as the absence of testes in a 46,XY individual with a male phenotype [1]–[3]. It affects one in 20,000 male births [4] and occurs in 1/177 cases of cryptorchidism [5]. Although some patients with anorchia present with ambiguous external genitalia [6] or microphallus [7], most have a normal phenotype. As the differentiation of the male genital tract and external genitalia is dependent on anti-Müllerian hormone (AMH) and testosterone, this suggests that functional testes were present, but disappeared *in utero* in these cases.

The familial occurrence of anorchia [1], [4], [6], [8] and its association with other anomalies [9] suggests a genetic origin, but the genetic cause remains unknown. The families of some patients with anorchia may include other individuals with pure or partial 46,XY gonadal dysgenesis. This has led to the suggestion that anorchia is part of the clinical spectrum of 46,XY gonadal dysgenesis [10]. However, exploratory laparoscopy has suggested that at least some cases of anorchia are the result of prenatal testicular vascular accident associated with torsion during testicle descent [11].

Testicle descent depends on the interaction between anatomical and hormonal factors. Ferlin et al [12] examined 600 boys with cryptorchidism and found 2 cases with a mutation in the gene encoding the protein required for correct testicle descent, insulin-like 3 (*INSL3*), and 5 cases with a mutation in the gene encoding its receptor (leucine-rich repeat-containing G protein-coupled receptor 8 (*LGR8*; also known as *GREAT*). We [13] have previously reported that in a cohort of 14 boys with bilateral anorchia mutations in the Y chromosome-linked testis-determining gene *SRY*, *INSL3*, and its receptor are not associated with anorchia. Others [14]–[16] also reported the absence of mutation in *SRY* gene. More recently, a novel heterozygous missense mutation in *NR5A1*, encoding steroidogenic factor 1 (SF1) gene, was found in a boy with a microphallus and testicle regression syndrome [17].

We have now extended our study of the 14 previous cases [13] to include 12 additional patients. The current study tests the efficacy of measuring plasma AMH and inhibin B concentrations as a diagnostic tool. Here we also report that in 7/26 families there were other family members with various anomalies of either the testes or ovaries, which led us to sequence genes involved in the development of both gonads and testicle descent.

## Materials and Methods

### Ethics statement

All the parents gave their written, informed consent for the evaluation, including chromosomal and molecular biology analyses, and for surgery. All clinical investigations were conducted according to the principals expressed in the Helsinki Declaration. The study was approved by the Ethical Review Committee (Comité de Protection des Personnes, Ile de France III).

### Patients

The patient cohort included 26 boys consecutively referred to one of us (R. Brauner) between 1981 and 2010 with anorchia (Table 1). DNA samples were available for all except 4 patients (cases 5, 15, 21 and 24). Anorchia was confirmed by the complete absence of testicular tissue at surgery, or by the presence of a small nodule of residual fibrous tissue. Cases 4, 6 and 11 were not operated on, but the clinical and biological data indicated that they also had anorchia. Surgery was performed by coelioscopy in cases 3, 7 and 20 and laparoscopy in the other 20. None of the patients had ambiguous external genitalia, hypospadias, or antecedents of radiotherapy, chemotherapy, testes trauma, inflammation, or infection. The family antecedents of abnormalities of the genitalia and fertility and previous palpations of testes by another physician were recorded. All the biological data were recorded before surgery and testosterone treatment.

**Table 1:** Characteristics of 26 boys with anorchia.

Patient	Testes at birth	Age at first evaluation, yr	Testostérone, ng/mL		AMH, pmol/L	Age at FSH,LH	FSH, IU/L		LH, IU/L		Age at surgery yr	Testes at surgery	Final height, cm
			Basal	After hCG			Basal	Peak	Basal	Peak			
1	R palpable. L not palpable	neonate	ND	ND	4	0.83	<0.4	8.7	<0.4	2.5	1.5	no tissue	
2	R+L palpable	0.7	<0.02	ND	2	ND	110	ND	ND	ND	3.5	no tissue	
3	R palpaple. L not palpable	1.2	0	ND	undetectable	1.2	81	155	4	125	1.5	bilateral résidues	
4	R+L not palpable	1.2	0	<0.05	2.1	1.2	107	285	20.5	203	NO	-	
5	R+L not palpable	1.2	ND	ND	ND	ND	ND	ND	ND	ND	ND	bilateral résidues	
6	R+L palpable	1.3	0	ND	2.5	1.1	68.2	ND	5.5	ND	NO	-	
7	R+L palpable	1.5	ND	ND	undetectable	1.5	102	ND	ND	ND	1	no tissue	
8	ND	2	<0.05	<0.05	undetectable	12.7	34	ND	6	ND	4.4	R no tissue. L résidue	178
9	neonatal bilateral torsion	3.2	0.2	ND	ND	13.2	87	ND	24	ND	0.5	bilateral résidues	184
10	R+L palpable	3.2	0	0	undetectable	7.1	13.3	ND	0.24	ND	4	bilateral résidues	
11	R+L not palpable	3.2	0	0.1	undetectable	3.4	57	149	0.72	22	NO	-	
12	R+L not palpable	3.5	ND	<0.1	undetectable	3.7	32	97	0.11	15	3.7	bilateral résidues	
13	ND	4.5	0	0	undetectable	6	18	77	<0.2	5.4	16	R no tissue. L résidue	183
14	R+L not palpable	4.8	0.36	ND	ND	13.2	6.3	ND	1.5	ND	4.8	bilateral résidues	>170
15	R+L palpable	5	<0.05	0.07	ND	12	9.4	ND	0.2	ND	ND	bilateral résidues	183
16	R+L palpable	5.1	ND	ND	undetectable	5.1	2	ND	<0.2	ND	ND	bilateral résidues	
17	ND	6.2	0.05	0.03	undetectable	6.2	0.6	10.9	<0.5	2.9	6	no tissue	
18	R palpaple. L not palpable	7.7	0	0	ND	11.8	106	ND	18	ND	8.6	R residue. L no tissue	
19	R palpaple. L not palpable	8	<0.05	0.1	ND	12.8	115	ND	32	ND	15.2	bilateral résidues	174
20	ND	8.2	ND	ND	undetectable	8.2	2.8	ND	ND	ND	7.7	no tissue	
21	R+L not palpable	8.7	<0.07	0.2	ND	12.5	61	ND	15	ND	8.6	R residue. L no tissue	175.6
22	ND	12.7	0.2	ND	ND	12.3	23	ND	12	ND	10.4	bilateral résidues	179
23	R+L palpable	12.8	0	ND	undetectable	12.8	120	ND	22	ND	8	R residue. L no tissu	167
24	R+L not palpable	13	<0.05	0.05	ND	13	49	ND	12	ND	7.7	no tissue	182
25	R+L palpable	13.6	0.09	ND	ND	13.6	122	ND	25	ND	15.2	R residue+L torsion	>180
26	R+L palpable	15.2	ND	ND	ND	15.8	64	ND	18	ND	9	bilateral résidues	172

ND, not determined; R, right; L, left; Inhibin B undetectable in cases 1, 2, 6, 7, 20 and 23.

The normal range values for FSH are 2–9 IU/L and for LH 1–5 IU/L.

### Methods

Leydig cell function was evaluated by measuring the plasma testosterone concentration before (n = 19) and after stimulation with human chorionic gonadotropin (hCG), (n = 12, 3 to 7 injections of 1,500 IU, given i.m. every other day, with samples taken the day after the last injection). Sertoli cell function was evaluated by measuring the basal plasma concentrations of AMH (n = 15) and inhibin B (n = 7) in the patients seen more recently. The basal plasma concentration of follicle stimulating hormone (FSH) was measured in 25 cases and luteinising hormone (LH) in 22 cases. The hypothalamo-pituitary-testicular axis was evaluated in 7 patients by measuring FSH and LH after stimulation with gonadotropin hormone releasing hormone (GnRH, 100 µg/m^2^). All plasma hormone concentrations were measured by RIA. When the assay method for a given hormone was changed during the study period, it was cross-correlated with the earlier method. Thus, the results are comparable throughout the study.

The complete open reading frames of *SRY*, *NR5A1*, *INSL3*, *MAMLD1* and the *T222P* variant for *LGR8* were sequenced as previously described [13], [18], [19].

## Results

### Clinical and biological data (Table 1)

#### No patient had any associated congenital anomaly

At birth, testes were palpable bilaterally in 9 cases, unilaterally in 4 cases and not palpable in 7 cases. In addition, case 9 presented with bilateral testicular torsion immediately after birth. The testes became unpalpable at 3 months in case 1, and before one year in cases 3 and 6. The age at which the other patients who had palpable testes at birth became unpalpable is unknown. At the first evaluation, all the patients had unpalpable testes and normal penis length and morphology, except case 1 who had microphallus and a small right testis palpable in the inguinal canal at birth with undetectable plasma inhibin B. He developed unexplained foot edema at 2 years.

The basal plasma concentrations of AMH, inhibin B and testosterone were very low or undetectable in all the patients evaluated, as were the increases in testosterone after hCG. The basal plasma concentrations of FSH were increased in 20/25, as was that of LH in 10/22 cases. The FSH after the GnRH test was increased in 5/7 patients, while that of LH was increased in 2/7 patients. The 5 patients with normal basal plasma FSH concentrations (cases 1,14,16,17 and 20) were aged 10 months to 13.2 years at evaluation.

Surgery was performed on 4 patients when they were aged 6 months to 2 years, on 12 patients when they were aged 2 to 9 years, and on 4 patients when they were older; information was not available for the other 3 patients (Table 1). Surgery showed no testicular tissue (6 cases), the presence of unilateral testicular residue (5 cases) or bilateral residues (12 cases, including unilateral torsion in case 25).

Testosterone treatment (25 mg i.m. every 14 days until the end of growth, then 250 mg every 21 days) was started for 14 patients between 12 and 15.9 years, taking into account their age at first evaluation, height and demand. Twelve reached their final height which was normal, as was their pubertal growth.

### Family history and associated malformations

#### Members of the families of 7 patients have gonadal anomalies

The mother of case 1 began menstruating at 12 years. She and the father of this patient had normal karyotypes. She was operated on for scalenus syndrome. She had a previous medical pregnancy interruption at 20 weeks for hygroma and anasarque. The female foetus had a 46,XX blood karyotype, intrauterine growth retardation, retrocervical edema, retrognatism, clinodactyly of the 5^th^ digit, agenesy of the 12^th^ pair of ribs, and ovaries with germ cells but no primordial follicles.

The mother of case 6 began menstruating at 9.5 years, then had irregular menstruations with increased basal plasma FSH concentrations and had 4 attempts at *in vitro* fertilizations before this pregnancy. The father had a normal spermogram. After the proband was born she underwent insemination, which led to an empty follicle. Her own mother became menopausal at 50 years and had a hysterectomy for fibroma.

#### The mother of case 25 entered the menopause at 28 years

The father of case 8 had secondary sterility with oligoasthenospermia. The father of case 13 had an increased basal plasma FSH concentration (12 IU/L) at 38 yr. The first cousins of cases 17 and 20 were operated for cryptorchidism.

Other significant clinical features of the patients included: the hair of case 5 became white when he was 20. The mother of case 7 had been treated for epilepsy by Depakine since the age of 12. The twin sister of case 23 began menstruating at 12.5 years and menstruates regularly at age 20.

### Genetic analyses

Analysis of the open-reading frames of the *SRY*, *NR5A1*, *INSL3*, *MAMLD1* and the *T222P* variant for *LGR8* revealed no mutations associated with the phenotype.

## Discussion

The present study documents several novel features associated with anorchia that have not been described previously. One is premature ovarian failure in two mothers and the absence of primordial follicles in the ovaries of one sister 46,XX after a medical pregnancy interruption for hygroma and anasarque. Two fathers had a tubular deficiency. As mutations involving the *NR5A1* gene have been associated disorders of sexual development (DSD) including anorchia [17], and primary ovarian insufficiency [19], we analysed the complete coding sequence of this gene. We also analysed the complete coding sequence of the genes involved in testicular descent because the first cousins of patients 17 and 20 had cryptorchidism. However, we did not detect any novel pathogenic variant of any of these genes, suggesting that other genes involved in the gonadal development contribute to these phenotypes.

### Clinical and biological data

One or both testes were palpable in 65% of the boys for whom information was available. Others have reported similar data [7], [20]. Zenaty et al [7] reported palpable intrascrotal or inguinal masses at first examination in 26/55 (47%) patients included in a multicenter study. However, they reported microphallus in 46% of patients, with similar proportions in boys with or without a palpable mass, whilst only one of our boys had microphallus. Rai et al [21] reported microphallus in three siblings with congenital anorchia.

How a testicular vascular accident and torsion during testicle descent is associated with anorchia is unclear. One of our patients suffered neonatal torsion, and it was previously reported in 10% of all cases [7]. Smith et al [11] studied 77 cases of testicular regression syndrome and found dystrophic calcification and haemosiderin deposits, with no evidence of viable testicular tissue, but with relatively normal spermatic cord elements. They stated that this supports the concept of unilateral or bilateral anorchia caused by a remote infarction due to torsion. Yerkes et al [22] surgically examined 18 patients for unilateral perinatal torsion and found contralateral torsion in 4 (22%); atrophy occurred in 4 cases despite orchidopexy of the better perfused gonad, and there were suggestions of contralateral torsion in 2 other cases.

The very low or undetectable plasma AMH and inhibin B concentrations seem to be a good biological marker of anorchia, as observed in our more recent patients and reported elsewhere. Lee et al [23] reported the same features in 12 children with anorchia. Kubini et al [24] reported that four of their patients with anorchia were clearly separated from those with abdominal testes, as they had undetectable inhibin B and no increase in plasma testosterone after hCG stimulation. They concluded that the diagnostic procedure may be reduced to a single inhibin B measurement in a boy with no palpable testes. Misra et al [25] concluded that an unmeasurable AMH concentration was 92.3% predictive of anorchia, while a low testosterone response to hCG was only 57.1% predictive.

The basal plasma FSH concentration is increased in boys with anorchia, but it may be normal and uninformative in patients aged between 2 years to pubertal age, as reported by De Rosa et al [26]. Our four patients with normal FSH concentrations included two whose testes had been palpable neonatally. This information was not available for other two, but their cousins had been operated on for cryptorchidism. These FSH concentrations increased when they reached pubertal age.

### Family history and associated malformations

Many reports have included patients with the 46,XY karyotype complement and agonadism associated with other anomalies and/or familial forms. The genitalia of these patients varied from a female appearance, ambiguous external genitalia, to normal male aspect and anorchia. We have focused on the reports that include at least one with a male aspect and anorchia.

Abeyaratne et al [1] and Bobrow and Gough [4] reported that two families each had one brother with bilateral and the other with unilateral anorchia. Josso and Briard [6] described two 46,XY agonadal siblings, one phenotypic male with microphallus and the other phenotypic female with slight fusion of the genital folds and no Müllerian ducts. They suggested that both disorders are due to regression of the embryonic testes. Naffah et al [8] reported the sibship of seven 46,XY individuals including three amenorrheic sisters and one brother with bilateral testis atrophy. Marcantonio et al [10] reported a family with nine 46,XY gonadal dysgenesis including one brother with anorchia, microphallus and no gonads, bilateral Fallopian tubes, and bilateral vas deferens. The others had ambiguous genitalia with no gonad (n = 3), or testes (n = 5) and one with *in situ* germ cell neoplasia. One had craniofacial and finger abnormalities and mildly retarded development, whilst another had multiple limb abnormalities. Rai et al [21] reported bilateral congenital anorchia in 3 siblings who were the products of nonconsanguineous marriage.

The families of our anorchia patients, included primary ovarian failure in two mothers, or a 46,XX sister with fetal malformations. The occurrence in the same family of 46,XY anorchia and gonadal abnormalities in 46,XX individuals has been described. Granat et al [27] reported finding two sisters 46,XX with gonadal dysgenesis, the brother was azoospermic and the parents consanguous. De Grouchy et al [9] reported finding embryonic testicular regression syndrome and severe mental retardation in three 46,XY siblings with varying degrees of genital ambiguity and another 46,XX with mental retardation. Kennerknecht et al [28] reported two phenotypic sisters with karyotype 46,XX and 46,XY, no Wolffian nor Müllerian derivatives, born to non-consanguous parents whose previous pregnancy ended in abortion intrauterine death of the embryo at 8 weeks of gestational age. Both had agonadism anad hypoplasia of the right pulmonary artery, hypoplasia of the right lung, isolated dextrocardia and diaphragmatic hernia or omphalocele. They suggested that the Y chromosome is not involved and that the disorder may be autosomal. Pierson et al [20] reported one patient with anorchia, a diaphragmatic hernia and aniridie, and another patient who, like his father had equilibrated translocation 3q−;20p+. This family included one patient with anorchia who was referred for infertility.

### Genetic analyses

We found no mutations associated with the phenotype. This confirms our previous finding [13] that mutations in *INSL3*, *SRY* and the *T222P* variant for *LGR8* are not associated with the phenotype, and suggest that *MAMLD1* mutations also do not contribute to the development of anorchia. It has been suggested that *NR5A1* is associated with bilateral anorchia, since one affected individual carried a heterozygous *NR5A1* mutation (p.V355M) [17]. However, we did not detect *NR5A1* mutations in the patients of our series suggesting that mutations in this gene are uncommon in anorchia. Thus other genetic factors appear to underlie the phenotype.

### Conclusion

Newborn apparently male babies with no palpable testes are most frequently diagnosed as cases of “simple” cryptorchidism. However, it is necessary to look for congenital adrenal hyperplasia presenting as Prader V and for a congenital hypothalamic-pituitary deficiency. Normal phallus size and morphology, and no hypoglycemia argue against these diagnoses, but an emergency assay of plasma 17α-hydroxyprogesterone is desirable and replacement therapy started to avoid a life threatening situation.

Anorchia is rare. Undetectable plasma concentrations of AMH and inhibin B and an elevated plasma FSH, together with 46,XY complement, are sufficient for its diagnosis. The hCG test is not necessary. Treatment with low doses of testosterone at pubertal age leads to a normal adult height.

We found gonadal deficiencies in two fathers and two mothers, and one sister had 46,XX and foetal malformations. The cousins of two patients suffered from cryptorchidism. But we detected no anomaly in a series of genes known to be involved in these conditions.
